# A Uniquely Challenging Case of Poorly Controlled Hyperthyroidism With a Coexisting Thyroglossal Cyst

**DOI:** 10.7759/cureus.14271

**Published:** 2021-04-03

**Authors:** Saeed Khalaf, Aysha Sarwani, Rawdha Al Fardan, Mohammed Maki, Mahmood Al Saeed

**Affiliations:** 1 Endocrinology, Salmaniya Medical Complex, Manama, BHR; 2 Internal Medicine, Salmaniya Medical Complex, Manama, BHR

**Keywords:** thyroglossal duct cyst, thyroid storm, thyroidectomy, graves' disease

## Abstract

Hyperthyroidism is a common disease. Thyroglossal duct cysts are also a very common type of congenital anomalies. Their co-existence is, however, rare with only a few cases described in the literature of the same. We describe the case of a 44-year-old female who presented initially 16 years ago with hyperthyroid symptoms related to Graves’ disease diagnosed serologically and by thyroid scan and ultrasound. Incidentally, she was also noted to have a non-complicated thyroglossal duct cyst. She was initially started on anti-thyroid medications but remained in a hyperthyroid state throughout most of her illness. Radioiodine ablative therapy and surgical resection were delayed due to the patient’s dispreference. After a long period of missed follow up, she presented with a thyroid storm and underwent a total thyroidectomy and cyst resection. The key endocrine issues learned from this case include: 1) dealing with non-compliant patients in terms of poorly controlled hyperthyroidism and refusal to follow the recommended treatment, 2) observing the natural disease progression of untreated Graves’ disease coexisting with a thyroglossal duct cyst, and 3) describing a rare incidental finding of a thyroglossal duct cyst coexisting with Graves' disease in the absence of ectopic hormone production. A few cases have described hyperthyroidism related to remnants of thyroid tissue in the thyroglossal duct or related to the thyroid gland. To the best of our knowledge, there are no cases reported of Graves’ disease coexisting with a non-complicated thyroglossal duct cyst making our case unique and first of its kind.

## Introduction

Thyrotoxicosis is a common disease with a female-to-male occurrence ratio of 5:1 [1]. Usually, patients present with the classical symptoms of hyperthyroidism including weight loss, heat intolerance, tremor, palpitations, anxiety, increased frequency of bowel movements, and shortness of breath. Goiter is commonly found on physical examination and, in most cases, it is related to Graves' disease. Thyroglossal duct cyst (TGDC) is the most common congenital anomaly in the neck [2]. TGDCs originate from the thyroglossal duct tract, which usually disappears between the 8th and 10th weeks of gestation. If, however, it did not disappear, thyroglossal duct epithelium will persist and it may form a cystic mass. Here, we present the case of a patient who presented with hyperthyroid symptoms related to Graves’ disease. What makes this case unique is the discovery of a coexisting non-functioning TGDC with no functioning ectopic thyroid tissue elsewhere. This, to our best of knowledge, has not been described in English literature before.

## Case presentation

A previously healthy female presented 16 years ago when she was 28 years of age to our endocrinology clinic with classic hyperthyroid symptoms including weight loss, palpitations, heat intolerance, and tremors. There was no significant family history of any illnesses. On examination, she was noted to be irritable with associated fine tremors and palmar erythema. Her eye examination revealed periorbital swelling with no associated lid lag, lid retraction or abnormal extraocular muscle movements. Her neck examination revealed a smooth and diffusely enlarged goiter with an associated bruit. Additionally, there was a lump noted nearby, which moved with tongue protrusion consistent with a TGDC. Vitally, there was associated tachycardia (120 beats-per-minute). Her laboratory investigations showed a T3 of 47 pmol/L, T4 of >100 pmol/L, and TSH of 0.00 microIU/L (Table 1).

**Table 1:** Timeline of the patient’s illness with biochemical laboratory results.

Year	Description	TSH (0.25-5.0) mIU/L	Free T3 (2.5-7.8) Pmol/L	Free T4 (6.0-24.5) pmol/L
2004	Initial presentation of hyperthyroidism. US Thyroid: Graves’ disease with Thyroglossal duct cyst. Carbimazole initiated.	0.00	47	>100
2004	Three months follow up: Improved symptoms. Carbimazole changed to Propylthiouracil due to side effects.	0.01	16	17
2005	Thyroid scan delayed due to pregnancy. Symptomatic throughout despite increasing PTU dose. Patient refused surgical treatment.	0.00	31.9	64
2008	Thyroid scan: Graves’ disease.			
2008-2013	Three uneventful pregnancies. Uncontrolled hyperthyroidism. Switched multiple times between propylthiouracil and carbimazole.			
2014	Trial of stopping anti-thyroid medications. Repeat thyroid scan: Grave’s disease. Patient refused radioiodine ablation and surgical resection. Carbimazole restarted.	0.00	-	14
2017-2019	Referred to the surgical team with pressure symptoms. Again, refused surgical intervention. Patient then lost follow up and stopped taking medications.	0.00	-	30
2020	Presented to the emergency department with thyrotoxicosis and heart failure. Agreed for surgical intervention. Uncomplicated total thyroidectomy and cyst excision performed.	0.00		>100
2020	Postoperatively: Controlled thyroid function on levo-thyroxine	0.2		13.9

**Figure 1:**
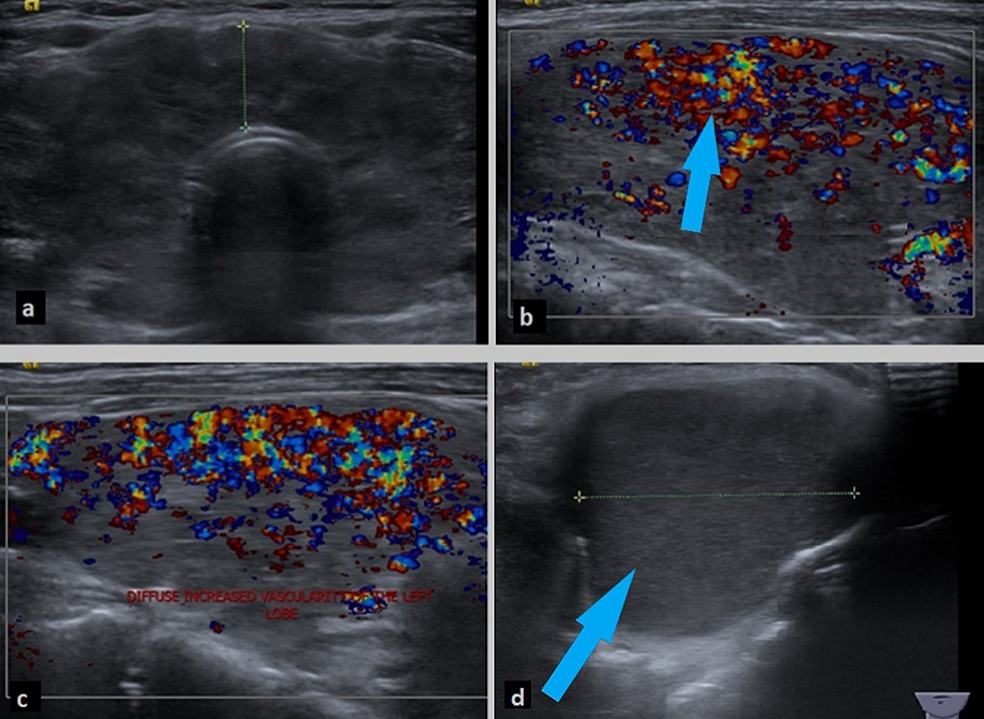
Ultrasonography of the thyroid gland showing an enlarged thyroid with heterogenous echotexture (a). Hypervascularity of the thyroid lobe consistent with thyroiditis noted by color Doppler of the right lobe (b) and left lobe (c). Also, large thyroglossal duct cyst measuring 4.4 x 3.3 cm in size is visible (d).

She was started on carbimazole 10 mg thrice daily and propranolol. A Technetium 99m pertechnetate thyroid scan and neck ultrasound were requested. She was reviewed in the clinic after three months and described improvement in her symptoms. She later improved with a T4 of 17 pmol/L, T3 of 16 pmol/L, and TSH of 0.01 microIU/L. Ultrasonography of her neck revealed an enlarged thyroid with heterogenous echotexture and hypervascularity in a Doppler study, consistent with thyroiditis. Also noted was an enlarged TGDC containing thick fluid. The cyst was 2.3x1.7 cm and was located at the base of the tongue at the midline with the possibility of a connection to the isthmus (Figures 1a-1d). She later complained of intolerance to carbimazole and was switched to propylthiouracil (PTU) 100 mg three times daily.

Before going for the thyroid scan, she became pregnant; therefore, the scan was rescheduled, and she was continued on PTU. Over the course of her pregnancy, her thyroid function tests (TFT) showed major fluctuations and she remained in a hyperthyroid state clinically and biochemically. She was, therefore, advised for surgery. After explaining the diagnosis and complications, she opted out of the surgical option and preferred to continue with medical therapy. She was kept on close monitoring but remained in a hyperthyroid state despite attempts of increasing the dosages of PTU.

A thyroid scan was eventually performed four years after her initial presentation due to multiple missed appointments and rescheduling. The scan revealed diffuse increased uptake of 17.6% (0.4%-4%), with no focal “hot” or “cold” areas. This led to the impression of a diffuse toxic goiter or Graves' disease.

Over the next four years, still reluctant to undergo surgery or radioiodine ablation, our patient got pregnant three times, all with successful deliveries. She was switched between carbimazole and PTU multiple times alongside very erratic TFT results. The surgical team advised her to undergo surgery several times, which was met with constant refusal.

**Figure 2:**
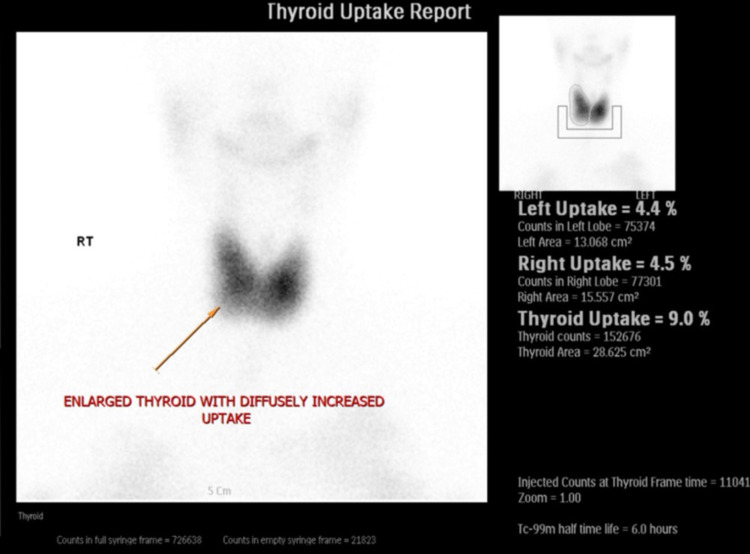
Tc-99m pertechnetate thyroid scan showing a diffusely enlarged thyroid gland with uptake of 9.0% (0.4%–4%) in a heterogenous distribution with no focal “hot” or “cold” areas.

Two years later, she was reviewed in the clinic and described no symptoms with a biochemical finding of subclinical hyperthyroidism. A repeat thyroid scan was done, which revealed an uptake of 9% and diffuse toxic goiter (Figure 2). She eventually developed pressure symptoms related to the goiter and was again advised for surgical resection, but she opted for medical management only. Unfortunately, she then lost follow up for several years.

Five months ago (16 years after initial presentation), she presented to the emergency department with palpitations, shortness of breath, presyncope, and confusion. Vitally, she was normotensive but tachycardic (140 beats-per-minute). On examination, there were audible bilateral lung crepitation and bipedal edema with marked ascites on abdominal examination. Her electrocardiogram showed atrial fibrillation and chest x-ray demonstrated pulmonary edema with pleural effusions. There was marked pulmonary hypertension and severe tricuspid regurgitation on echocardiography. She was started on furosemide, metoprolol, and carbimazole. Computed tomography of her abdomen was arranged, which showed features of heart failure as a complication of uncontrolled hyperthyroidism. There was marked cardiomegaly, a dilated inferior vena cava, liver congestion, and moderate ascites. The surgical team was re-involved for definitive treatment and this time, the patient agreed to go for a total thyroidectomy and cyst resection. The procedure was uneventful with no immediate complications. She was then started on levo-thyroxine and her TFTs have been stable ever since with complete resolution of her symptoms.

**Figure 3:**
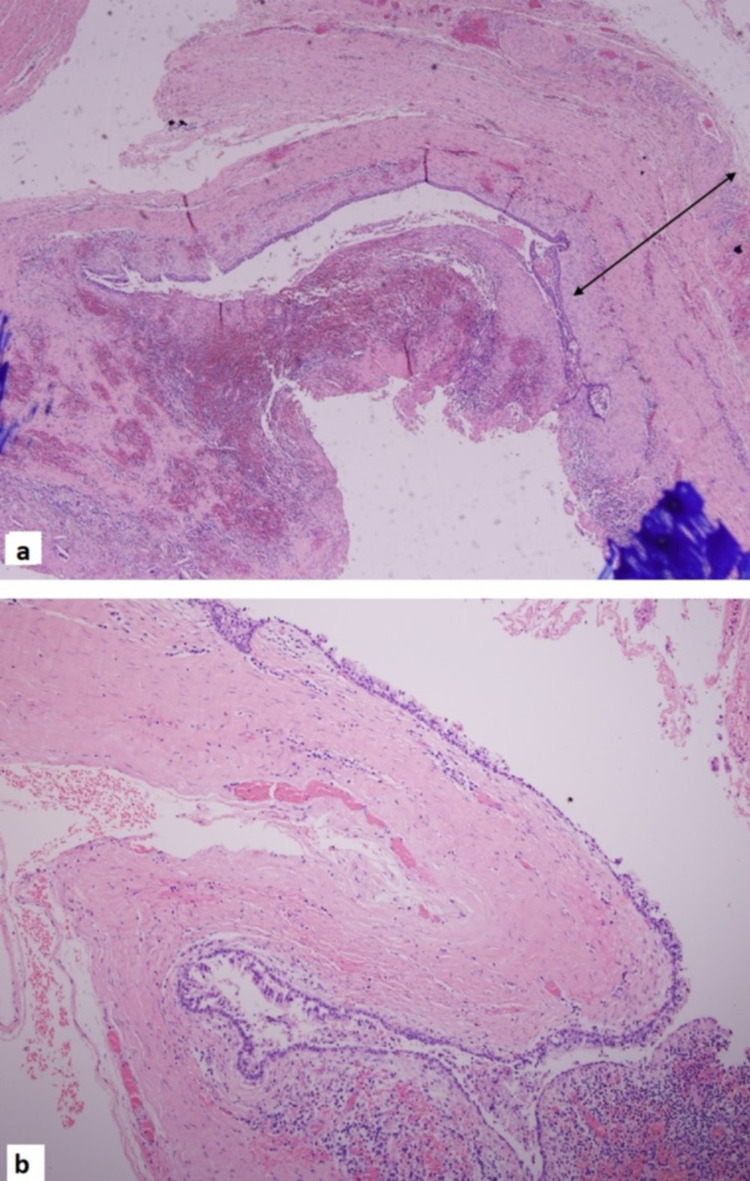
Hematoxylin and eosin (H&E) stain showing the thyroglossal cyst wall with focal ulceration and inflammation x5 magnification (a). H&E stain of the thyroglossal cyst wall lined by respiratory epithelium x10 magnification (b).

There were two excised specimens - the thyroid gland and the TGDC. The cystic lesion of 5.5 x 3 x 0.5 cm was excised with no abnormal gross pathology. Both the thyroid lobes were excised with no abnormal gross pathology. Histology of the thyroid gland was consistent with lymphocytic thyroiditis. The TGDC specimen was histologically consistent with an inflamed TGDC (Figures 3a, 3b).

## Discussion

Thyrotoxicosis is a common disease with a female predominance. When diagnosing hyperthyroidism, the combination of clinical presentation, clinical examination, and biochemical findings should be taken into consideration [2,3]. The classic clinical presentation consists of heat intolerance, palpitations, tremor, weight loss, and anxiety. Clinical examination findings include ocular manifestations, moist and warm skin, tachycardia, hyper-reflexia, and hyperactivity. In terms of biochemistry, the classical findings include low serum TSH and high free T4 and/or T3 concentrations. Radiological investigations are also very important including radioiodine uptake in non-pregnant women. Alternatively, in pregnancy or breastfeeding, the assessment of thyroidal blood flow on ultrasonography is acceptable to distinguish Graves' disease from other causes of hyperthyroidism [4]. This is evident in our case where ultrasonography demonstrated generalized hypervascularity. Treatment of hyperthyroidism is dependent on the case, ranging from medical therapy to radioiodine ablation therapy or complete surgical resection [5].

TGDCs are considered one of the commonest cases of neck masses in young children [6]. Normally, the cyst disappears by the 10th week of gestation; however, sometimes, remnants of the tract and thyroid tissue remain and enlarge, leading to a cyst [7,8]. TGDCs can be found at any location along the embryologic course of the thyroid gland [9]. The commonest site of ectopic thyroid tissue is the lingual thyroid with the second commonest being the wall of TGDCs [9]. Almost all ectopic thyroid tissue reside in TGDCs alongside a normally developed thyroid gland at its usual location [10]. Classically, TGDCs present in children or adolescents and are usually asymptomatic but can lead to numerous complications such as infection, malignancy, and pressure symptoms. Therefore, surgical removal is sometimes necessary once the patient is deemed fit [11].

Our case describes hyperthyroidism associated with a normal TGDC which is extremely rare. Only a few cases of TGDC with abnormal TFTs are reported in the literature. All those cases have the source of thyrotoxicosis originating from the TGDC. Our case is unique as the source of thyrotoxicosis originated from the thyroid gland while the TGDC was not responsible.

Buckingham et al. reported a case of Graves' disease in the cervical thyroid and thyroglossal duct remnant where the focus of increased thyroid activity was in the remnant TGDC in addition to the thyroid gland [12]. Basili et al. reported a case of recurrence of Graves' disease in thyroglossal duct remnants presenting as relapse after a total thyroidectomy [13]. Their patient developed clinical signs of hyperthyroidism post thyroidectomy and a thyroid scan revealed an area of increased uptake in the thyroglossal duct. In another case reported by Rao et al., a 49-year-old man, while being investigated for Graves' disease, was found to have a diffuse toxic goiter and a radiotracer concentration in the thyroglossal duct on Tc-99m pertechnetate scan [14]. Finally, Chopra et al. described the case of a 13-year-old girl presenting with thyrotoxicosis due to thyroiditis in the thyroid gland in addition to the thyroglossal duct as an ectopic thyroid source [15].

To the best of our knowledge, all cases described in English literature describe thyroglossal duct ectopia being the source of hyperthyroidism on top of the thyroid gland. On the other hand, our case demonstrated a focus of increased uptake at the thyroid gland only.

Our case describes a non-compliant patient with a complicated outcome. Thyroid storm and heart failure are well-recognized complications of untreated thyrotoxicosis. One of the most important endocrine emergencies is thyroid storm. Although rare and affecting only 10% of all cases admitted with thyrotoxicosis, it carries a high mortality rate of up to 30% [16]. The complications of thyrotoxicosis are multi-systemic and can lead to multi-organ failure most notably, the cardiovascular system. Thyrotoxicosis can present with tachycardia, atrial fibrillation, cardiomyopathy, accelerated hypertension, and pulmonary edema. Congestive heart failure occurs in up to 6% of patients with thyrotoxicosis [17]. In most cases, the resulting heart disease is related to uncontrolled atrial fibrillation and prolonged significant tachycardia. It usually resolves once sinus rhythm is restored or the tachycardia is controlled.

Our patient constantly refused to follow medical and surgical advice. Such non-compliant behavior interferes with the effectiveness of therapy and may lead to serious consequences. When dealing with such cases, it is of paramount importance to identify the stem of the problem whether it is failure of communication and understanding, lack of trust, or patients’ fears. It is therefore important to counsel those patients and try to identify the reason for the non-compliant behavior. Physicians must also make sure such patients understand all the details of their illness and the consequences of non-compliance. Constant reminders of the situation and regular discussions are the key to overcome such challenge.

## Conclusions

Long-standing uncontrolled hyperthyroidism treated with oral medications may lead to numerous complications. Definite treatment with radioactive iodine ablation therapy or surgical removal is needed in such patients. Co-existent ectopic hyperfunctioning thyroid cells in the thyroglossal duct have been reported previously, but normal TGDC tissue and Graves' disease with a goiter has never been reported. Non-compliance and refusal to follow medical advice can make cases challenging. It is therefore important to closely follow these cases and make sure patients have clearly understood the risks and outcomes. After a long 16-year history of uncontrolled hyperthyroidism, surgery with complete excision of the thyroid gland and TGDC was performed in our patient, which was completely curative.
